# Unraveling the complexities of early-onset colorectal cancer: a perspective on dietary and microbial influences

**DOI:** 10.3389/fpubh.2024.1370108

**Published:** 2024-04-04

**Authors:** Axelle Mayode Atchade, Jennie L. Williams, Linda Mermelstein, Barbara Nemesure

**Affiliations:** ^1^Stony Brook Medicine, Stony Brook, NY, United States; ^2^Cancer Center, Stony Brook Medicine, Stony Brook, NY, United States

**Keywords:** early onset colorectal cancer, gut microbiome, westernized diet, microbial influence, antibiotic use

## Abstract

While advances in screening have resulted in declining rates of colorectal cancer (CRC) among adults ≥50 years of age since the mid-2000s, the incidence of early-onset CRC (EOCRC) has steadily increased over the last decade. This increase is not fully accounted for by hereditary factors, and the hypothesis that a sedentary lifestyle and obesity are the primary culprits is not fully supported by recent reports indicating that many affected individuals lead active lifestyles, maintain normal weight, and are otherwise healthy. Attention has shifted toward dietary patterns, notably the consumption of processed and ultra-processed foods found in Western diets, which are suspected of disrupting the gut microbiome balance that potentially leads to EOCRC. The impact of antibiotic use on the gut microbiome is also posited as a contributing factor, given its rising prevalence in medical and agricultural practices. We propose that a paradigm shift is necessary for EOCRC research, moving beyond metabolic factors to a broader exploration of dietary and microbial influences. Future research must prioritize understanding the relationship between dietary habits, particularly processed food intake, antibiotic exposure, and gut microbiome dynamics, to unravel the complex etiology of EOCRC. This will be crucial in developing comprehensive preventive strategies to address the increasing incidence of this malignancy in younger populations.

## 1 Introduction

Colorectal cancer (CRC) is the third most common cancer diagnosis worldwide and ranks second in cancer-related mortality in the United States (1, 2). Notably, CRC takes on a particularly alarming role as the leading cause of death among men under 50 years of age on a global scale. Even with advances in screening, a 1–2% annual increase in incidence rates has been witnessed among young adults since the mid-1990s, resulting in a shift from 11 to 20% of cases occurring in individuals under 55 years from 1995 to 2019 (2, 3).

Genetic mutations such as Lynch Syndrome and Familial Adenomatous Polyposis contribute to approximately 1/4 of CRC diagnoses (4–7), but these hereditary syndromes do not appear to explain the onset of cancer in the majority of patients under the age of 50 years (8, 9). Additionally, there has not been an indication to date that the incidence of these germline mutations has increased over time. While lifestyle factors and the obesity epidemic have been proposed as drivers for increases in CRC among younger patients (10–12), a striking number of cases occur in individuals lacking metabolic syndromes, or those without attributable risk factors such as smoking or consumption of alcohol. Furthermore, these cases are often diagnosed at advanced stages with poor cell differentiation (3, 7, 13). This prompts a cascade of questions surrounding the unidentified mechanisms and factors contributing to these increased incidence rates in this demographic. Herein, we explore the intricate connection between the surge in EOCRC and the factors influencing its pathogenesis, such as metabolic syndromes, processed foods, antibiotics, and the colonic microbiome.

## 2 The influence of metabolic syndrome

To assess the influence of metabolic factors on CRC, a retrospective study was undertaken using data obtained from the Stony Brook Cancer Registry, which records demographic, lifestyle, clinical, and other factors for all cancer cases (of any type) diagnosed at The Stony Brook University Hospital (SBUH). The evaluation included more than 900 CRC patients diagnosed at SBUH between 2010 and 2020 and evaluated factors such as gender, race, age and year at diagnosis, marital status, family history of cancer, smoking status (current, former, never), and history of alcohol consumption (current, former, never). Descriptive statistics stratified by age at diagnosis were used to quantify the distribution of all risk factors under evaluation, and statistically significant associations were defined as those with *p*-values <0.05. Eleven percent of patients were under the age of 50 years at the time of diagnosis, and the data indicated that most cases were not obese, nor did they have a history of diabetes, hypertension, or hyperlipidemia (Table 1). Furthermore, these prevailing metabolic factors, often implicated in CRC risk, exhibited a disparate pattern, with rates being more than four times lower in CRC cases under 50 compared to those aged 50 and above.

**Table 1:** Characteristics of *N* = 909 patients with colorectal cancer stratified by age at diagnosis.

Characteristics	<50 Years (*n* = 101)	> = 50 Years (*n* = 808)	*p*-value
Gender, % Male	56.4	54.6	0.75
Race, %			0.55
Black	8.9	7.3	
White	91.1	92.7	
Family Hx Cancer, %	65.5	57.7	0.17
Smoking Status, %			<0.01
Current	21.6	15.7	
Former	13.4	44.7	
Never	65.0	39.6	
Alcohol Consumption, %			0.09
Current	45.3	45.6	
Former	2.1	8.1	
Never	52.6	46.3	
Marital Status, %			<0.01
Single	44.4	14.2	
Married	45.5	54.8	
Divorced/Separated	10.1	31.0	
Diabetes Hx, %	4.0	18.2	<0.01
Hypertension Hx, %	10.9	49.9	<0.01
Obesity Hx, %	10.9	16.6	0.15
Hyperlipidemia Hx, %	4.0	26.7	<0.01

Similarly, in a study reported by Chen et al., including 253 early-onset CRC cases, only 3.6 and 5.5% exhibited overweight and obesity, respectively, in stark contrast to the 13.4 and 5.6% observed in cases aged 50 years and above (4). These findings further dispel the notion that obesity in younger patients serves as a primary driver for the escalating incidence rates within this demographic. Of additional note, these factors are increasing in the population as a whole, thereby making it unclear why such metabolic influences would only raise incidence among younger patients while rates continue to decline among those ≥50 years old who have a larger burden of compromised health (12).

Traditional lifestyle factors such as cigarette smoking and alcohol consumption also fail to account for the rise in early-onset CRC cases. As shown in Table 1, more than half of the cases under 50 years reported never consuming alcohol, and 65% never smoked. Additionally, the percentage of abstainers among younger patients tended to exceed that of their older counterparts. The conventional links between these lifestyle behaviors and CRC incidence appear elusive in the context of early-onset disease.

In light of these observations, the focus shifts toward the gut microbiome as a potential orchestrator of the increasing trend in CRC among those under 50 years. Alterations in the microbiota, induced by various factors such as environmental exposures, antibiotics, sedentary lifestyle, and dietary intake, emerge as plausible contributors. Given the significant changes in the American diet over the past several decades, as well as findings from a recent worldwide systematic review including 12 countries and 5 continents, which indicated that increasing CRC risk in younger adults is being driven by rising rectal cancers in North America and Australia (14), we postulate that dietary shifts may be pivotal in disrupting the gut microbiome. These disruptions, in turn, may foster adverse cellular changes in the gastrointestinal tract, providing a novel perspective on the intricate web of influences contributing to EOCRC incidence. A closer inspection of the evolution of dietary consumption patterns in the US since the 1900’s may help to elucidate mechanisms responsible for noted disruptions in the gut microbiota.

### 2.1 The dietary shift

**Figure 1:**
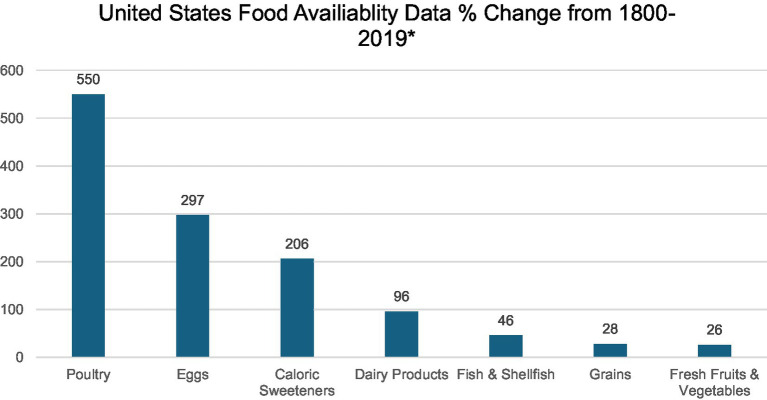
*Data extracted from Lee et al.’s study on United States Dietary Trends Since 1800 (15).

During the past century, the American diet has undergone radical changes, marked by a notable increase in the consumption of processed and ultra-processed foods, including refined carbohydrates, sugar, white flour, white rice, and industrial seed/vegetable oils (15, 16). This evolution is not merely a shift in dietary preferences but a journey into the unknown landscape of the Westernized colonic microbiome that is breeding a silent epidemic of EOCRC in young adults. The dietary evolution is characterized by a 206% surge in caloric sweeteners and has witnessed a staggering 550% increase in the availability of poultry from 1800 to 2019 (15) (Figure 1). The rise of industrial seed oils and vegetable shortening, triumphing over traditional animal fats, mirrors a broader trend in the infiltration of ultra-processed foods, such as chips, ready-to-eat meals, pre-packaged snacks, cereals, hot dogs, and energy bars, into more than 50% of the American diet.

The story unfolds further in the realm of meat processing, a domain increasingly marked by chemical preservatives and alterations in saturated fatty acid sources, resulting in colonic inflammation. Meat processing methods (i.e., curing, salting, smoking, and canning) result in high amounts of saturated fats, trans fats, and cholesterol (16). The curing process, for example, results in the release of nitrates and nitrites, endogenous N-nitroso compounds (NOCs), (16, 17) further cementing the link between dietary choices and CRC. In addition, studies show regular red meat consumption which contains heme induces toxicity and is a catalyst for epithelial damage, compensatory hyperproliferation, and eventual hyperplasia, a precursor to CRC. The repercussions extend beyond the immediate realm of meat consumption. Cooking methods, particularly at high temperatures, transform innocuous meats into carcinogenic agents, releasing polycyclic aromatic hydrocarbons (PAHs) and heterocyclic amines (HCAs) (16, 17). This toxic union with NOCs triggers mutations in pro-inflammatory genes, stimulates DNA damage through alkylation (16, 17), and sets the stage for tumorigenesis.

Liang et al. further cast a spotlight on the Western-style diet, reporting a culinary preference among the young compared to older individuals residing in Taiwan, who consume a more traditional Taiwanese diet (18). This dietary preference emerged as a potent force causing genetic or epigenetic alterations leading to microsatellite instability, a precursor to CRC (5, 19). Increases in EOCRC were primarily driven by distal colon and rectal tumors between 2004 and 2013 (20), and this trend was highlighted through the study by Zheng et al., revealing stronger associations between diet quality and early-onset advanced adenomas in the distal colon and rectum compared to the proximal colon (20). The intricacies of tumor progression further reveal that the proximal CRC is more likely to advance through the serrated neoplasia pathway, which contrasts with the majority of distal cancers originating from conventional adenomas. These distal cancers bear the molecular signatures of APC and TP53 mutations (20).

The molecular composition becomes a key player, as prior analyses in older cohorts expose the stronghold of the Western dietary pattern on tumors with specific molecular characteristics. These characteristics include low MSS or microsatellite instability, non-CpG island methylator phenotype, and BRAF/KRAS wild type which are representative of the molecular subtype common in EOCRC (20, 21). Collectively, the evidence points to a compelling conclusion that diet may wield a more potent influence on neoplasia originating from the traditional adenoma-carcinoma sequence (20), a stark reminder of the intricate interplay between dietary choices and the molecular landscape of colorectal cancer. However, while changes in food availability and patterns of consumption are believed to yield a significant impact on gut health, it is not likely that diet alone is responsible for the noted increases in EOCRC incidence. Additional factors such as medications, exposures and other potentially negative influences require further consideration.

### 2.2 Colonic microbiota and antibiotics

The colonic microbiota, a complex ecosystem profoundly influenced by factors such as diet, toxins, antibiotics, and pathogens, emerges as a critical player in the development of CRC. Among these factors, enteric pathogens pose the greatest risk of causing microbial imbalance, thus initiating or worsening tumorigenesis through mechanisms such as chronic inflammation, immune suppression, and the production of cancer-promoting metabolites (16). Dietary choices, particularly those high in meat and animal fat, have been identified as significant contributors to dysbiosis, fueling the production of genotoxic hydrogen sulfide (H_2_S) and the secretion of bile acids, which metabolize into carcinogenic secondary bile acids (19, 22). Notably, distinctions between low-risk and high-risk CRC populations have emerged, with the latter exhibiting an overabundance of proinflammatory bacteria, including *Porphyromonas, Fusobacterium, Enterococcaceae, and Bacteroides-Prevotella* genera, while beneficial and short-chain fatty acid-producing bacteria like *Faecalibacterium prausnitziin*, known for its anti-inflammatory properties, are diminished (22). Research demonstrates that the gut microbiota’s sulfur metabolism, particularly influenced by Western dietary habits, plays a direct role in carcinogenesis (23). Notably, H_2_S exhibits dual effects, depending on its origin and concentration. High levels can damage the mucosa by disrupting disulfide bonds in the mucus layer, allowing luminal bacteria and their byproducts to breach the epithelial lining, triggering apoptosis and inflammation (23), especially in regions where sulfur-metabolizing bacteria are abundant, such as the distal colon. Interestingly, research suggests that low concentrations of H_2_S, both endogenous and from exogenous dietary sources like garlic and cruciferous vegetables, can protect and repair the colonic epithelium by promoting vasorelaxation, reducing stress, and preventing cell death (23).

Additionally, research has shown that in the United States, African Americans carry the biggest burden of CRC, and many have hypothesized that this could be due to diet-induced changes in the microbiome. Indeed, the incidence of colon cancer in rural South Africans has been reported to be substantially less than that for African Americans (24). O’Keefe et al. compared changes within the mucosal membrane of African Americans and rural South Africans following a dietary exchange. This was conducted to test the theory that Western diets contribute to increased CRC incidence (24). The rural South Africans were provided with a high-fat, low-fiber Westernized Diet for two weeks (25), whereas the African Americans ate the low-fat, high-fiber African-style diet. Alterations in colonic mucosal biomarkers, microbiota (e.g., *Fusobacterium nucleatum*), and metabolome found in the colon of South Africans were linked to increased risk of CRC (across all ages) (24, 25). The alternative, a decrease in risk factors, was observed for African Americans on the South African-style diet. Taking into account the emergence of EOCRC, one could reasonably speculate that exposure to risk factors, such as a Western diet high in ultra-processed foods during childhood and adolescence, could contribute to increased incidence rates. Since a considerable time lapse is required for normal colonic mucosa to develop into cancer, significant physiological and metabolic disturbances beginning in early life may partially account for the rising incidence of sporadic EOCRC (23, 26).

As we examine all age groups, a pattern of decreased microbial diversity and dysbiosis emerges as a common denominator in CRC risk. Pediatric studies hint at the early-life origins of dysbiosis as the setting for EOCRC development, linking it to factors like mode of delivery and antibiotic exposure during critical developmental periods (26, 27). While causative links between EOCRC and microbiota remain elusive, studies establish a positive correlation between the use of anti-anaerobic antibiotics and colorectal cancer. The gut microbiome, predominantly composed of anaerobes, appears susceptible to dysbiosis induced by anti-anaerobic antibiotic interventions, potentially fostering an environment conducive to colorectal tumor growth. The medical field’s reliance on antibiotics, crucial for treating bacterial infections, introduces a paradox. The collateral damage inflicted on beneficial short-chain fatty acid-producing gut bacteria raises concerns about antibiotics’ unintended consequences, particularly in the context of EOCRC. In the 1980s, the use of broad-spectrum antibiotics in the US tripled due to inappropriate prescriptions for ear and upper respiratory tract infections in children (6, 13, 27). The historical surge in antibiotic prescriptions, especially in pediatric populations, underscores the need for judicious antibiotic use to mitigate the risk of dysbiosis and its potential contribution to EOCRC. Furthermore, disparities in antibiotic prescription rates have indicated that White children received more antibiotics than Black children (27, 28), raising the question of how race factors into the colonic microbiome. Racial disparities in EOCRC incidence rates further complicate the narrative. While incidence rates for non-Hispanic Blacks have seen a marginal increase, the incidence rates for Hispanics and non-Hispanic Whites have surged to 85% within the same time span (4). The role of race and antibiotic exposure in shaping the colonic microbiome merits deeper exploration to untangle the complex web of contributing factors.

Beyond medicine, the extensive use of antibiotics in agriculture poses an additional threat to the gut microbiome. Antibiotics are employed in livestock for growth promotion and disease prevention, potentially leading to the transfer of antibiotic-resistant bacteria from animals to humans (3). The United States Food and Drug Administration (FDA) prohibited the use of antibiotics for growth promotion in 2017 and required that medically important antibiotics be prescribed by a veterinarian (29). However, these guidelines are non-binding and voluntary, and there are loopholes that allow the continued use of medically important antibiotics in agriculture. The ramifications for public health and nutrition are compounded by the conditions of livestock and factory farms. For example, the prevalent use of constricted battery cages for laying hens fosters unsanitary conditions and disease, both in poultry and potentially in their eggs (29). This is particularly disconcerting considering the staggering 550% increase in poultry food availability over the last century (Figure 1). Pesticides on fresh produce and the presence of toxic contaminants in fish-inhabited waters represent additional factors contributing to changes in the gut microbiome. Additionally, the modern diet, loaded with additives like artificial coloring and preservatives, further compounds the issue by influencing the gut microbiome. Exogenous factors, including diet and environmental exposures, intricately shape microbial diversity, consequently impacting metabolism, immune responses, and gene expression (6).

### 2.3 Discussion

The rising incidence of EOCRC poses a significant and alarming public health challenge. While previous research has predominantly linked metabolic syndromes to this trend, our investigation unveils a more intricate narrative, emphasizing the role of changing dietary patterns, particularly in Westernized societies. The traditional focus on factors such as obesity, diabetes, and hypertension fail to account for over half of EOCRC cases, urging a reevaluation of the multifaceted contributors to this growing issue. This realization underscores the need for a broader exploration of potential contributors, leading us to scrutinize the significant role that dietary patterns and lifestyle behaviors play in the context of EOCRC.

One pivotal aspect of this discussion revolves around the profound changes in human nutrition witnessed over the past century. The shift toward diets rich in meat, fats, oils, and added sugars, coupled with reduced consumption of vegetables and whole grains, has created an environment conducive to inflammatory processes within the intestinal microenvironment (30). While the exact mechanisms remain elusive, the proposed link between inflammatory diets and EOCRC highlights the importance of understanding the metabolic decomposition of lipids, particularly secondary bile acids and hydrogen sulfide (16), as potential instigators of inflammation and subsequent damage to the intestinal epithelial barrier.

A notable consequence of dietary transformation is the decline in fiber intake, primarily due to increased consumption of refined grains and sugars (31, 32). This decline has been associated with an elevated risk of colon cancer, emphasizing the critical role of fiber in maintaining intestinal health. The statistical evidence of a substantial increase in the availability of caloric sweeteners and sugar-sweetened beverage consumption further underscores the urgency of addressing excessive sugar intake despite some recent declines (22, 30, 32, 33).

The protective potential of a healthy diet, characterized by a high intake of fruits, vegetables, legumes, whole grains, and low-fat dairy (22), is underscored by epidemiological studies revealing an inverse relationship between colorectal adenomas and carcinomas and fiber intake (34). Encouragingly, these findings suggest that increased fiber intake during childhood and adolescence may serve as a protective measure against EOCRC.

The evaluation of the surge in EOCRC consistently points to changes in Westernized dietary patterns in the United States as a significant contributor. These dietary alterations, in turn, impact the colonic microbiome, adding a layer of complexity to our understanding of EOCRC etiology. Importantly, these changes in the microbiome persist even in the absence of traditional metabolic syndromes. Such findings warrant further research into the intricate relationships between diet, gut microbiota, and initiation of cancer (16). A shift in focus from traditional metabolic factors to dietary patterns, coupled with efforts to regulate processed foods and promote judicious antibiotic use, is crucial. This includes targeted interventions and therapies aimed at inhibiting incidences of EOCRC. Public health campaigns and collaborative efforts among researchers, healthcare professionals, policymakers, and the public are imperative to reverse this trend and improve gastrointestinal health in younger generations. As we advance in our understanding of these relationships, we pave the way for informed strategies that can effectively mitigate the risk of EOCRC and promote better overall gastrointestinal health.

## Data availability statement

The original contributions presented in the study are included in the article/supplementary material, further inquiries can be directed to the corresponding authors.

## Ethics statement

The studies involving humans were approved by Stony Brook University Institutional Review Board. The studies were conducted in accordance with the local legislation and institutional requirements. Written informed consent for participation was not required from the participants or the participants’ legal guardians/next of kin because the data were retrospective and fully de-identified.

## Author contributions

AMA: Data curation, Formal analysis, Investigation, Methodology, Writing – original draft, Writing – review & editing. JW: Conceptualization, Investigation, Methodology, Supervision, Writing – review & editing. LM: Conceptualization, Data curation, Methodology, Writing – review & editing. BN: Conceptualization, Data curation, Formal analysis, Investigation, Methodology, Project administration, Supervision, Validation, Writing – original draft, Writing – review & editing.
